# Optimizing Clinical Implementation of Hypofractionation: Comprehensive Evidence Synthesis and Practical Guidelines for Low- and Middle-Income Settings

**DOI:** 10.3390/cancers16030539

**Published:** 2024-01-26

**Authors:** Maria Thereza Mansur Starling, Stephane Thibodeau, Cecília Félix Penido Mendes de Sousa, Felipe Cicci Farinha Restini, Gustavo A. Viani, Andre G. Gouveia, Lucas C. Mendez, Gustavo Nader Marta, Fabio Ynoe Moraes

**Affiliations:** 1 Division of Radiation Oncology, Department of Oncology, London Health Sciences Centre, London, ON N6A 5W9, Canada; 2 Division of Radiation Oncology, Department of Oncology, Kingston General Hospital, Queen’s University, Kingston, ON K7L 3N6, Canada; 3 Johns Hopkins Bloomberg School of Public Health, Baltimore, MD 21205, USA; 4 Radiation Oncology Department, Hospital Sirio Libanês, Sao Paulo 01308-050, Brazil; 5 Department of Medical Imagings, Ribeirão Preto Medical School, Hematology and Oncology of University of São Paulo (FMRP-USP), Ribeirão Preto 14049-900, Brazil; 6 Latin America Cooperative Oncology Group (LACOG), Porto Alegre 90619-900, Brazil; 7 Division of Radiation Oncology, Department of Oncology, Juravinski Cancer Centre, McMaster University, Hamilton, ON L8V 5C2, Canada

**Keywords:** hypofractionated radiotherapy, stereotactic body radiation therapy, low- and middle-income countries (LMICs), treatment duration optimization, resource-limited settings

## Abstract

**Simple Summary:**

The increasing global cancer burden is intensifying health disparities, especially in low- and middle-income countries (LMICs), where access to advanced treatments is limited. A notable challenge in these regions is the scarcity of radiation therapy services, crucial for cancer treatment. This research review explores hypofractionated radiotherapy (HRT) and ultra-hypofractionated/stereotactic body radiation therapy (SBRT) as promising alternatives, offering shorter treatment durations and optimizing the use of radiotherapy machines. These methods are particularly beneficial for LMICs, addressing the growing demand for cancer care. This review provides updated clinical evidence and guidelines for applying HRT and SBRT in treating various cancers, highlighting the need for rigorous training, advanced technology, and strict safety protocols. It also emphasizes the importance of comprehensive support and collaboration to enhance access to these advanced cancer treatments in resource-limited settings.

**Abstract:**

The global cancer burden, especially in low- and middle-income countries (LMICs), worsens existing disparities, amplified by the rising costs of advanced treatments. The shortage of radiation therapy (RT) services is a significant issue in LMICs. Extended conventional treatment regimens pose significant challenges, especially in resource-limited settings. Hypofractionated radiotherapy (HRT) and ultra-hypofractionated/stereotactic body radiation therapy (SBRT) offer promising alternatives by shortening treatment durations. This approach optimizes the utilization of radiotherapy machines, making them more effective in meeting the growing demand for cancer care. Adopting HRT/SBRT holds significant potential, especially in LMICs. This review provides the latest clinical evidence and guideline recommendations for the application of HRT/SBRT in the treatment of breast, prostate, and lung cancers. It emphasizes the critical importance of rigorous training, technology, stringent quality assurance, and safety protocols to ensure precise and secure treatments. Additionally, it addresses practical considerations for implementing these treatments in LMICs, highlighting the need for comprehensive support and collaboration to enhance patient access to advanced cancer care.

## 1. Introduction

Cancer poses a global public health impact, with an estimated 20 million new cases and 10 million deaths in 2020 worldwide [1]. In the next two decades, the burden of cancer is projected to increase significantly, placing additional strain on healthcare systems, individuals, and communities [2].

Low- and middle-income countries (LMICs) already comprise approximately 60% of cancer cases and the majority of cancer death [2,3]. However, while 88% of high-income countries (HICs) have access to such registries, they are available in only 45% of LMICs, which makes the issue ever more challenging. This hampers an accurate assessment of local epidemiology, evaluation of the demands, appropriate planning and capacity building, and access to curative cancer treatment (surgery, systemic therapy, and radiotherapy) [2,4].

Radiotherapy (RT) is a major component of cancer treatment, and almost 50% of patients with cancer will require RT at some point during their treatment path [5]. However, only 62% of countries report having RT services readily accessible [4]. The current global shortage of RT infrastructure and investment hinders the achievement of accessible, timely, and high-quality RT [6,7,8].

According to the International Atomic Energy Agency Directory of RT Centers database [9], HICs have a ratio of one RT machine per 120,000 people, while middle-income countries have one for over 1 million people. In low-income countries, a single machine serves approximately 5 million people [9]. LMICs would need to increase RT availability by at least tenfold to approach the same level of access in HICs [3,4]. This shortage, coupled with the rise in the burden of cancer, requires urgent attention [3,10,11,12,13].

Hypofractionated RT (HRT) has emerged as a viable solution for tackling the scarcity of RT, particularly in LMICS. HRT involves administering a higher radiation dose with each session (fraction) using either moderate (>2–4 Gy per fraction) or ultra-hypofractionation (>5 Gy per fraction) [14], thereby reducing the overall duration of RT [10,15,16]. From a radiobiological standpoint, hypofractionation offers many therapeutic benefits, especially for tumors characterized by low α/β ratios [17]. In such cases, larger fractions can lead to a proportionally higher dose delivered to the tumor [17,18] as compared to conventional fractionation (1.8–2 Gy per fraction). The ESTRO-GIRO survey indicates a strong preference for hypofractionation in treating bone metastasis, with more than 85% of respondents choosing this approach. It is also preferred for node-negative breast cancer post-lumpectomy (82.2% utilization), while it is less commonly used for node-positive breast cancer (46.7%, *p* < 0.001). Similarly, hypofractionation is more frequently utilized for low- and intermediate-risk prostate cancer (57.5% and 54.5%, respectively), as compared to high-risk prostate cancer (41.2%, *p* < 0.001) [19].

This review systematically evaluates the clinical evidence supporting the use of hypofractionation and ultra-hypofractionation in the treatment of prostate, breast, and lung cancers. It is designed to function as an educational resource, guiding physicians in the effective application of these techniques. This is particularly crucial for low- and middle-income countries (LMICs), where resource limitations pose unique challenges.

In alignment with the broader theme of this edition, which includes detailed discussions on gynecological and head and neck cancers in other articles, our focus is on these three cancer types. Through a literature search, we sought relevant studies that could enrich our understanding of hypofractionation’s role in LMICs. However, we found that while there are non-prospective studies from LMICs that offer some insights, there were no prospective single-arm studies or clinical trials from LMICs that significantly enhanced the context and technological considerations beyond what our existing references already cover. To maintain the article’s focus and avoid redundancy, we chose to include only those studies that substantially contribute to the discussion.

In this review, we present critical evidence and provide recommendations for managing advanced stages of prostate, breast, and lung cancers, integrating the most recent findings from key 2023 conferences like ASTRO and ESTRO. We also incorporate relevant guidelines in the field. This approach ensures that our review remains a current, comprehensive, and practical guide for practitioners, especially in LMIC settings where adapting to the latest in cancer treatment is vital yet challenging.

## 2. Required Minimum Infrastructure for Hypofractionated Radiotherapy Adoption in LMICs

Utilizing HRT necessitates advanced technology, meticulous treatment planning, delivery, and stringent quality assurance due to its increased dose per fraction to the target volume.

A notable disparity in the adoption of HRT, particularly in LMICs, can be attributed to substantial gaps in technology and infrastructure. Although not mandatory, certain prerequisites are recommended for safely implementing HRT. These include a linear accelerator (LINAC) for three-dimensional conformal radiation therapy (3DCRT), CT treatment simulation with sub-3 mm slice reconstruction, both forward and inverse treatment planning software, proper immobilization devices, a comprehensive QA protocol, and a team of well-trained radiation oncology professionals [16,20,21,22].

While these are the minimum standards for safely and adequately delivering HRT, the preferred treatment delivery method involves volumetric modulated arc therapy (VMAT) or intensity-modulated RT (IMRT) utilizing a minimum 6 MV beam, accompanied by onboard image guidance and motion tracking systems [16]. In addition, regardless of the chosen technique (3DCRT, IMRT, or VMAT), certain QA tasks should be performed more frequently than the standard LINAC QA guidelines of the Task Group 142 report [23].

In certain clinical cases, safe treatment can be administered to patients without the need for advanced techniques, thanks to the surrounding normal tissue. Though modern guidelines typically advocate for at least 3D treatment for enhanced safety and better protection of normal tissue, the absence of technology should not be a barrier to adopting HRT, as long as the existing technology ensures safe treatment delivery. In a phase II clinical trial conducted in India, a two-week hypofractionation regimen was employed for patients diagnosed with breast cancer, utilizing a two-dimensional conformal radiation therapy (2DCRT) treatment technique. The study demonstrated favorable outcomes, with acceptable late toxicities, commendable cosmesis, and excellent local control [24]. However, for prostate and lung cases, which we will discuss the clinical evidence of in the upcoming sections of this article, our recommendation is to utilize a minimum of 3D technology, emphasizing concerns related to associated toxicity.

The high-dose requirements of HRT and SBRT necessitate strict safety measures for organs at risk (OAR), calling for precise margins around the target and advanced image guidance strategies beyond conventional radiographic methods. This demands substantial resources including specialized personnel, technology, and time. It is crucial to establish clear guidelines, ensure staff certification, provide ongoing training, and implement comprehensive quality management and QA procedures at multiple intervals, to secure safe and effective treatment delivery [24,25,26]. For the details of the safe implementation, it is advisable to consult the ASTRO Safety White Paper Update [25].

Accurate patient positioning and image quality in treatments like SBRT necessitate the use of appropriate localization tools (e.g., cone beam CT or other 3D imaging methods) and rigorous, ongoing QA procedures, overseen by a qualified physicist during every treatment session. Daily validations of the image-to-accelerator alignment and regular end-to-end tests for new imaging technologies are crucial. Strict adherence to the guidelines outlined in the American Association of Physicists in Medicine Task Group Report 101 is imperative to ensure the safety and effectiveness of the treatment [27]. These recommendations can also be applied to HRT to accommodate the increased dose rate.

## 3. Clinical Evidence for Hypofractionated Radiotherapy

### 3.1. Breast

Breast cancer is the most common malignancy globally [28,29]. In 2020, breast cancer diagnoses reached 2.3 million women, resulting in 685,000 deaths worldwide. GLOBOCAN’s predictions show a concerning increase in breast cancer. By 2040, cases are expected to rise by more than 45%, leading to around 3 million new cases each year, and breast cancer-related deaths are anticipated to go up by more than 50%, from 685,000 in 2020 to 1 million in 2040 [29,30].

Following surgical interventions like mastectomy or lumpectomy, postoperative radiotherapy (RT) is vital in the comprehensive care of breast cancer patients, significantly lowering the risks of recurrence and breast cancer-related deaths [31,32]. Implementing HRT for breast cancer in LMICs has the potential to optimize resource utilization and possesses a well-established radiobiological advantage [33,34]. Studies suggest that increasing the daily fraction size while reducing the total dose might offer equal tumor control to conventional fractionation (CF), with a theoretical small trade-off of late toxicity [34,35,36]. As a result, HRT has been widely embraced, promising better outcomes for patients with breast cancer around the world [17,37,38].

#### 3.1.1. Evidence Supporting Moderate Hypofractionation in Breast Cancer

Moderate HRT to the breast yields local control and survival outcomes that are at least as effective as those achieved with CF and has become the standard regimen for whole-breast irradiation (WBI) after breast-conserving surgery or mastectomy in the vast majority of women [39,40]. Previously published meta-analyses have thoroughly analyzed various aspects of its effectiveness [41]. Ten-year outcomes were reported in four identified trials: the UK’s START trials -P, -A, and -B, along with the Ontario Clinical Oncology Group (OCOG) trial from Canada [34,42,43,44]. The START -P and -A trials were three-arm trials that examined two experimental HRT schedules over 5 weeks. Conversely, both START-B and the OCOG trials were pragmatic trials that tested 15 or 16 daily fractions of 2.7 Gy over 3 weeks, comparing them with the historical standard of 50 Gy in 25 fractions daily over 5 weeks. The OCOG trial included 1234 women with T1-2 N0 M0 breast cancer who had undergone breast-conserving surgery followed by CF-WBI or HRT-WBI. The 10-year results showed that HRT-WBI was not inferior, with no discernible differences in the skin or subcutaneous tissue observed in late adverse events [43,45].

The Beijing trial involved 820 pT3–4 pN2–3 post-mastectomy breast cancer patients who were randomly assigned to receive post-mastectomy radiotherapy (PMRT) of the chest wall and nodal irradiation (supraclavicular and level 3) at 50 Gy in 25 fractions over 5 weeks or a 3-week hypofractionation regimen delivering 43.5 Gy in 15 fractions. The main objective was to compare the local control between the conventional dose and hypofractionation groups, and after 5 years, there were no significant differences in locoregional relapse rates. Additionally, both acute and late toxicities were comparable between the groups, with the hypofractionated radiotherapy group experiencing less severe acute skin toxicity (3% vs. 8%; *p* < 0.001) [46].

Meta-analysis studies found no statistically significant differences between the two treatment approaches including in locoregional recurrence, disease-free survival, distant metastasis, and overall survival [41,47,48,49,50]. Also, there were no significant differences in acute and late skin toxicity, acute lung toxicity, lymphedema, shoulder restriction, and late cardiac-related toxicity between HRT and CF [47,50]. Overall, acute, and late side effects tended to be lower after HRT compared to conventional fractionation, such as breast shrinkage, distortion, induration, and shoulder stiffness. One trial indicated slightly better cosmesis [51] in the HRT group, while other studies found no difference in cosmetic outcomes [43,52]. Long-term results [34,42,43,44,53] support the use of HRT as a preferred alternative to the traditional 2 Gy daily fraction. A 2022 systematic review encompassed breast cancer patients ranging from ductal carcinoma in situ (DCIS) to more advanced stages, undergoing various treatments and randomly assigned to either HRT or CF. While there was no specific analysis for these subgroups, the evidence strongly suggests that moderately hypofractionated irradiation is beneficial for these patients [41].

For cases of DCIS, there is randomized evidence supporting the consideration of HRT as the standard [52,54], with endorsements from ESTRO and the NCCN Guidelines. As demonstrated by recent randomized findings from the DBCG HYPO Trial, the use of moderately hypofractionated breast irradiation in cases of node-negative breast cancer or DCIS did not lead to an elevated occurrence of breast induration in comparison to standard fractionated therapy. Moreover, the impact on surrounding normal tissues was minimal, with similar or less frequent effects observed in the 40-Gy group. Furthermore, the risk of locoregional recurrence over the course of 9 years remained notably low [52]. At the 2023 ASTRO annual meeting, a study involving 385 women with stage 0–III breast cancer compared HRT to CF after mastectomy with implant-based breast reconstruction. The results showed similar recurrence rates and no link between fractionation type and chest wall toxicity, highlighting the viability of hypofractionation in post-mastectomy breast reconstruction scenarios [55].

#### 3.1.2. Evidence Supporting Ultra-Hypofractionation in Breast Cancer

Ultra-hypofractionated WBI has emerged as a notable and viable treatment option for early-stage breast cancers. This approach has acquired considerable attention for its ability to provide comparable oncologic and toxicity outcomes while also offering the added benefits of enhanced patient convenience and improved radiotherapy compliance because treatment is delivered over an even shorter time than with moderate HRT.

Ultra-hypofractionation in 28.5 Gy in five fractions (once-a-week) may be considered for selected patients over 50 years following breast-conserving surgery with early-stage, node-negative disease. This evidence emerged from the ten-year results of the tumor and side-effect endpoints reported in the FAST trial, which showed no significant difference in normal tissue effects and disease outcomes [56].

The FAST-Forward trial, a randomized phase 3 non-inferiority study, explored an ultra-hypofractionated treatment regimen of 26 Gy or 27 Gy in five fractions delivered within a week for patients with pT1–3, pN0–1, and M0 status after mastectomy or breast-conserving surgery. After a median follow-up of almost 6 years, the results indicated that this accelerated treatment approach was non-inferior to the standard 40 Gy in 15 fractions over 3 weeks for ipsilateral breast tumor relapse. Additionally, the ultra-hypofractionated schedule of 26 Gy demonstrated comparable safety in terms of normal tissue effects (whereas 27 Gy was worse). Data beyond 5 years regarding local relapse or toxicity are currently unavailable, though the trend-line in survival may actually favor the hypofractionated regimen, while late toxicity could still manifest on longer follow-up [57]. Some consensus guidelines recommend ultra-hypofractionation in select circumstances based on the data available to date. The characteristics of some included trials that assessed hypofractionation and ultra-hypofractionation in breast cancer are detailed in Table 1, Table 2 and Table 3.

#### 3.1.3. Current Guidelines and Consensus Recommendations

In 2018, the ASTRO guidelines [58] recommended two specific hypofractionation (HRT) schemes for women with invasive breast cancer undergoing whole-breast irradiation (WBI), irrespective of various tumor and patient characteristics. Age and factors like tumor grade, chemotherapy, hormone receptor status, and HER2 receptor were deemed irrelevant in deciding to offer HRT. HRT was also suggested as an alternative for patients with DCIS. The guidelines emphasized minimizing breast tissue exposure above the prescription dose but did not provide clear recommendations on HRT for regional lymph nodes and the chest wall. Nevertheless, a subsequent randomized trial indicated that HRT post-mastectomy, including regional lymph node and chest wall irradiation, is as effective and safe as conventional fractionation (CF), with no significant difference in late complications [46].

In this context, we highly recommend incorporating the recent update into everyday clinical practice. The European Society for Radiotherapy and Oncology’s advisory committee and the St Gallen 2021 consensus both strongly advocate for the use of hypofractionated radiation therapy (HRT) across a broad range of breast cancer scenarios, including whole-breast and chest wall irradiation, as well as regional nodal irradiation (RNI), regardless of age at breast cancer diagnosis, pathological tumor stage, breast cancer biology, surgical margins status, tumor bed boost, breast size, invasive or pre-invasive ductal carcinoma in situ (DCIS) disease, oncoplastic breast-conserving surgery, and use of systemic therapy [40]. These schedules remain highly endorsed for patients with breast reconstructions after mastectomy as well [59].

While ultra-hypofractionation, a regimen of 26 Gy in five fractions [57], is gaining interest, the St. Gallen Panel has not established it as a standard treatment yet. However, recent ESTRO guidelines have embraced this approach for whole-breast irradiation (WBI) and chest wall irradiation, particularly without breast reconstruction, or within a clinical trial or registered cohort. That said, practitioners are urged to proceed with caution when applying ultra-hypofractionation to chest wall irradiation post-breast reconstruction and to nodal irradiation.

The HYPORT-Adjuvant trial, a phase III study, is currently comparing the effectiveness of two radiation treatments for high-risk patients. It is examining if a 3-week schedule of 40 Gy in 15 fractions is as good as a 1-week schedule of 26 Gy in 5 fractions, including regional radiotherapy for both. Initial findings show a higher rate of grade 2/3 radiation dermatitis in the 3-week schedule (11.1%) than in the 1-week schedule (2.9%). The trial is still in progress and continuing to recruit patients [60]. The interim analysis indicates that ultra-hypofractionation is feasible for acute toxicity management, but it should be restricted to randomized trials or registered cohorts until findings from ongoing studies are available [40,59].

### 3.2. Prostate

Based on the 2020 GLOBOCAN data, prostate cancer ranks as the second most prevalent cancer in men worldwide and accounts for approximately 375,304 cancer deaths [28,61]. RT is a well-established curative option for localized diseases, with its efficacy robustly supported by numerous randomized trials. These studies have consistently shown that administering cumulative doses ranging from 75.6 to 79.2 Gy to the entire prostate gland significantly decreases biochemical recurrence and enhances metastasis-free survival [62,63,64,65,66].

Prostate cancer, much like breast cancer, demonstrates a unique radiobiological feature with its low α/β ratio, generally estimated to be about 1.5 Gy [67]. On the flip side, the α/β ratio for adjacent healthy tissues is roughly 3.0 Gy, and even higher for the rectum at around 5.0 Gy [68]. This significant difference (low α/β tissues are known to be more sensitive to fraction size than high α/β tissues) underscores the critical necessity for accurate and precise radiation therapy, ensuring optimal targeting of cancer cells while sparing the neighboring healthy tissues to the greatest extent possible [17,18]. Employing HRT in this context could simultaneously enhance the radiobiologically equivalent tumor dose without resulting in increasing the prevalence of treatment-induced toxicity [17,18].

Technological advancements in radiation therapy, including 3DCRT, IMRT, and VMAT, along with daily image guidance (IGRT), have greatly enhanced treatment precision for prostate cancer. This has significantly reduced radiation doses to critical organs like the rectum and bladder, paving the way for extensive research and numerous clinical trials aimed at identifying the most effective hypofractionated radiation therapy (HRT) regimens and optimal dose per fraction [69,70,71,72,73].

#### 3.2.1. Evidence Supporting Moderate Hypofractionation in Prostate Cancer

Patients with non-metastatic prostate cancer should be considered for moderately HRT with image-guided regimens involving 2.4–4 Gy per fraction delivered over 4–6 weeks. These treatment schemes have been extensively evaluated in large randomized trials that have cumulatively enrolled over 6000 patients, and their effectiveness has been shown to produce similar or non-inferior results compared to CF [69,71,72,73,74,75].

The optimal regimen for HRT to the prostate has not been established. Several schemes are endorsed and supported by the largest evidence base, including 60 Gy in 20 fractions and 70 Gy in 28 fractions, with or without the inclusion of seminal vesicles and the omission of pelvic lymph nodes [73,75,76,77]. The CHHiP trial randomized 3216 men with predominantly intermediate-risk disease into three unique RT arms. At a median follow-up of 5.2 years, it demonstrated that 60 Gy in 20 fractions had non-inferior biochemical and clinical failure rates compared to the 74 Gy CF regimen [73,76]. In patient-reported outcomes up to 5 years after treatment, there was no significant difference in bowel, urinary, and sexual symptoms between the treatment schedules, thereby reinforcing the consistently low occurrence of moderate/high bother [78]. The PROFIT trial investigated a moderately HRT image-guided approach for 1206 men with intermediate-risk prostate cancer. Comparing two treatment arms, the hypofractionated regimen with 60 Gy in 20 fractions demonstrated non-inferiority in biochemical–clinical failure rates compared to the CF arm with 78 Gy in 39 fractions over 7.8 weeks [79].

A meta-analysis of ten phase III trials showed that HRT provides outcomes comparable to CF, but with increased acute gastrointestinal (GI) toxicity. Subgroup analysis revealed that the use of androgen deprivation therapy and the extent of seminal vesicles included in the treatment volume significantly influenced acute GI toxicity rates during HRT. Patients not using androgen deprivation therapy and those with a smaller treatment volume of seminal vesicles experienced notably fewer instances of acute GI toxicity [80]. Similar results—comparable oncologic outcomes, including overall survival and prostate cancer-specific survival and a higher incidence of acute GI toxicity—were also shown in a Cochrane meta-analysis comprising 10 randomized controlled trials with 8278 patients [81].

#### 3.2.2. Evidence Supporting Ultra-Hypofractionation in Prostate Cancer

Prostate ultra-hypofractionation and SBRT (generally called in this section SBRT) has strong evidence to support its use for treating localized prostate cancer, especially in men with low- and intermediate-risk disease who do not need nodal irradiation. Numerous phase 1 to 3 trials have consistently shown excellent disease control with minimal toxicity [82].

The phase III HYPO-RT-PC trial demonstrated that 42.7 Gy in 7 fractions (every other day) was as effective as 78.0 Gy in 39 fractions for patients with intermediate-to-high-risk prostate cancer regarding freedom from failure. The study followed patients for a median of 5 years and found that at the 5-year mark, SBRT was just as effective as CF at controlling biochemical recurrence and late side effects, though we await the long-term results for more conclusive evidence. Patients reported experiencing more early side effects with SBRT, and by the end of the treatment, physician-scored grade 2 or worse genitourinary (GU) toxicity was slightly higher in the SBRT group (28% versus 23%). However, there were no significant increases in late, grade 2, or worse GU or bowel side effects, except for a minor rise in urinary toxicity one year after SBRT (6% versus 2%, *p* = 0.004) [83]. However, it is crucial to emphasize that a subsequent analysis of patient-reported quality of life (QoL) indicated that SBRT was as well tolerated as CF up to six years after treatment [84].

In the phase III non-inferiority PACE-B trial, 874 men with low- or intermediate-risk prostate cancer, excluding Gleason 4 + 3, were randomly assigned to two treatment arms: CF with 78 Gy in 39 fractions over 7.8 weeks/HRT with 62 Gy in 20 fractions over 4 weeks (following a protocol amendment), or SBRT with 36.25 Gy in 5 fractions over 1–2 weeks. The eligibility criteria included prostate cancer T1c-T2c, Gleason score ≤ 3 + 4, PSA (ng/mL) ≤ 20, MRI staging, and no androgen deprivation therapy (ADT), and the primary outcome of PACE-B was to assess freedom from biochemical or clinical failure. This trial demonstrated no significant difference between the groups in short-term toxicity rates [85]. Additionally, the 5-year results presented at the 2023 ASTRO Annual Meeting were comparable, with no serious adverse events reported within the analyzed timeframe. SBRT demonstrated a biochemical–clinical failure event-free rate of 95.7% (95% confidence interval [CI] = 93.2–97.3%), whereas CF/HRT exhibited a rate of 94.6% (95% CI = 91.9–96.4%). These outcomes confirmed the non-inferiority of SBRT to conventional radiotherapy (90% CI, *p* for non-inferiority = 0.007). Adverse event rates were low in both groups and did not significantly differ between the treatment arms. At the 5-year post-treatment mark, 5.5% of SBRT recipients experienced grade ≥ 2 side effects, compared to 3.2% in the conventional group (*p* = 0.14). Only one patient in each group experienced grade ≥ 2 gastrointestinal side effects (*p* = 0.99) [86].

A comprehensive meta-analysis and systematic review examined 38 prospective series, comprising 6116 patients, over an average follow-up period of 39 months (12 to 115 months). Among the included studies, 92% of patients were classified as low-risk, 78% as intermediate-risk, and 38% as high-risk. The overall rates of biochemical relapse-free survival at 5 and 7 years were found to be 95.3% and 93.7%, respectively. The estimated rates of significant GU and GI toxicity occurring later in the treatment were 2.0% and 1.1%, respectively, and within 2 years after SBRT, urinary and bowel functions returned to baseline. The meta-analysis also revealed that increasing the dose of SBRT was associated with enhanced biochemical control (*p* = 0.018) but led to worse late-grade 3 GU toxicity (*p* = 0.014). These findings provide strong evidence supporting the use of SBRT for localized prostate cancer, showcasing excellent disease control, minimal toxicity, and negligible effects on QoL. Notably, these results primarily pertain to patients with low- and intermediate-risk diseases and may not be directly applicable to those with high-risk [82].

In summary, substantial evidence supports using SBRT for treating localized prostate cancer in low- and intermediate-risk patients, showing it is safe and effective. However, more data are needed for high-risk patients to ensure safe application. To fully grasp its benefits and risks compared to other treatments, we need long-term results from randomized trials, which are crucial for conditions like prostate cancer that can have late failures. The characteristics of some included trials that assessed hypofractionation and SBRT in prostate cancer are detailed in Table 4 and Table 5, respectively.

#### 3.2.3. Current Guidelines and Consensus Recommendations

According to the ASTRO/ASCO/AUA evidence-based guideline on HRT for localized prostate cancer, there is low-quality evidence supporting the use of SBRT for intermediate- and high-risk cases. However, they do acknowledge that SBRT may be considered for men with low- and intermediate-risk disease if their prostate size is less than 100 cm^3^. The guideline recommends offering SBRT as part of a clinical trial or multi-institutional registry. It is important to note that this guideline was based on a systematic literature review of studies published between 1 December 2001 and 31 March 2017 [70]. The National Comprehensive Cancer Network (NCCN) Version 1.2023 advocates for the possibility of using SBRT for all risk categories of prostate cancer, provided that appropriate expertise and technology are available [87].

### 3.3. Non-Small Cell Lung Cancer (NSCLC)

Lung cancer is a global health issue, with about 2.2 million new cases and 1.79 million deaths annually, making it one of the most frequently diagnosed and leading causes of cancer-related mortality worldwide [28,88]. This highlights the urgent need for ongoing research, prevention efforts, and improved treatment strategies to address this challenging disease.

Lung cancer treatment involves a complex mix of surgery, radiation therapy (RT), systemic treatments, interventional radiology, and palliative care. RT is unique as it is applicable to all stages of lung cancer and varied patient conditions. Data suggest that around 77% of lung cancer patients may need RT during their treatment, with HRT and SBRT becoming increasingly important across all disease stages [89,90].

#### 3.3.1. Evidence Supporting Moderate Hypofractionation in NSCLC

Resection, with or without adjuvant systemic therapy, is considered the standard of care for operable patients diagnosed with stage I/II NSCLC [91,92]. In medically or surgically inoperable patients, SBRT is considered the standard of care (see next section). While SBRT is an excellent alternative in many patients, it requires excellent immobilization and planning resources, which may not be available in all contexts where RT is available. In early-stage disease, proceeding to an aggressive course of treatment with concurrent chemoradiation would be the only alternative; however, HRT plays an important role in potentially obviating the need for systemic therapy in this setting [93,94].

Abstract data from the LUSTRE trial stand as the largest phase III trial encompassing patients with stage I medically inoperable NSCLC, where patients were randomized to receive either SBRT of 48 Gy in 4 fractions (peripheral) or 60 Gy in 8 fractions (central—within 1 cm of mediastinum or 2 cm of the proximal bronchial tree), versus HRT of 60 Gy in 15 fractions. With 233 patients recruited, SBRT showed a trend towards improved local control (LC) compared to HRT (87.6% vs. 81.2%), but the trial was underpowered to confirm this (*p* = 0.15). No significant differences were observed in disease-free survival (*p* = 0.40) and overall survival (*p* = 0.40) and severe late toxicities were rare. When SBRT is unavailable, acceptable HRT schedules may encompass either 20 fractions of 2.5–3.3 Gy [93] or 15 fractions of 4 Gy [94]. So, in cases where surgery is not an option for inoperable patients, SBRT serves as an alternative, while HRT can also be considered for medically inoperable patients. However, it is important to note that the LUSTRE trial revealed differences between these two modalities. If a well-established SBRT program is not accessible, HRT becomes a viable alternative for patients who are medically inoperable.

In the context of inoperable locally advanced NSCLC, the most effective curative option involves concurrent chemoradiation with CF, followed by immunotherapy in those who do not progress [95,96]. However, it is crucial to acknowledge that some patients with NSCLC may not be suitable candidates for concurrent chemoradiation due to medical comorbidities or cancer-associated decline in performance status, where treatment-related toxic effects would outweigh potential benefits. In such cases, HRT remains a valuable alternative alone or in patients who could be candidates for sequential chemotherapy [97,98].

A comprehensive analysis of a large national radiotherapy data set compared outcomes of two commonly used regimens: the HRT regimen (55 Gy in 20 fractions) and the CF regimen (60–66 Gy in 30–33 fractions). The study included 12,898 patients with NSCLC, revealing that patients treated with the moderate HRT regimen had worse survival outcomes, even after correction for significant prognostic factors such as age, stage, comorbidity, and use of surgery. Specifically, patients treated with the 2.75 Gy per fraction regimen had a median survival of 25 months, compared to 29 months for those treated with the 2 Gy per fraction regimen (HR = 1.16, *p* = 0.001) [99]. However, the absence of randomization is likely to introduce some selection bias and residual confounding that could overstate the benefit of CF when HRT is more often used in patients who expressly cannot tolerate more aggressive therapy.

In a randomized phase II trial, patients with inoperable stage III NSCLC and good performance status were randomly assigned to receive cisplatin and vinorelbine with either sequential or concurrent chemoradiation using HRT with 55 Gy in 20 fractions over four weeks. Treatment-related mortality was 2.9% and 1.7% for the concurrent and sequential groups, respectively, with a relative risk (RR) of 1.25 (95% CI 0.55, 2.84). Toxicity was similar between arms. HRT combined with chemotherapy sequentially or concurrently is feasible, reasonably safe, and shows promising outcomes [98].

A recent phase III trial investigated HRT in poor-performance-status patients with stage II/III NSCLC who could not receive concurrent chemoradiation to determine whether it could improve overall survival. Eligible patients were randomized to HRT (60 Gy in 15 fractions) versus CF (60 Gy in 30 fractions). The study failed to reveal a survival benefit in an interim analysis, and the trial was prematurely closed due to futility. It is worth mentioning as a limitation that the outcomes may have differed in an immunotherapy setting. However, in a subgroup analysis of patients, there was a trend towards improvement with HRT in time to local recurrence and distant metastasis, resulting in a trend of fewer patients dying of NSCLC. Nonetheless, there was an almost 3-fold increase in grade 2 toxic effects overall [97]. Because the trial was designed to assess the superiority of HRT and was not powered to show equivalence, HRT should be considered with caution.

A meta-analysis examined the efficacy and safety of HRT combined with chemotherapy in the context of NSCLC. The study aimed to assess outcomes such as overall mortality, local failure, and disease progression in patients treated with concurrent chemotherapy and HRT versus sequential chemotherapy followed by HRT. An indirect comparison was made with the standard treatments, including concurrent and sequential CF chemoradiation. Based on two randomized controlled trials involving 288 patients, the results indicated no significant differences in overall mortality, disease progression, or local failure at 3 years between HRT with concurrent chemotherapy and HRT with sequential chemotherapy. Furthermore, late-grade 3 pneumonitis and esophagitis showed no significant variation. In the indirect comparison with conventional treatments, the outcomes for the HRT arms were also similar. These findings suggest that HRT, combined with chemotherapy, can yield favorable results in locally advanced NSCLC and should be considered for inclusion in future clinical trials, alongside exploring innovative strategies such as immunotherapy [100].

In conclusion, HRT stands as a viable treatment option for carefully selected patients with comorbidities and reduced performance status. Physicians should carefully consider individual patient factors and weigh the benefits and potential toxicities when deciding on the appropriate treatment strategy. Further research and randomized controlled trials are warranted to better elucidate the efficacy and safety of HRT in this clinical setting.

#### 3.3.2. Evidence Supporting Ultra-Hypofractionation/SBRT in NSCLC

In patients with inoperable T1/T2 N0 NSCLC, SBRT is considered the standard of care. Pioneering Japanese research on SBRT for early-stage lung cancer demonstrated remarkable results, revealing very low toxicity rates (2.4% grade 3 or higher) and excellent local control (over 90%) in patients treated with doses achieving BED ≥ 100 Gy [101].

SBRT was formally compared with CF/HRT. The phase III CHISEL trial demonstrated that SBRT significantly reduced local treatment failures in medically inoperable stage I NSCLC patients compared to CF. The SBRT group had a treatment failure rate of 14% vs. 31% in the standard RT group (*p* = 0.01). Moreover, SBRT led to improved survival, with 5-year survival observed compared to 3 years in standard RT (HR 0.53, 95% CI 0.30–0.94) [93]. The balanced distribution of staging processes, patient characteristics, and tumor factors in the CHISEL trial solidified its status as a landmark study that demonstrated the superiority of SBRT and established it as the standard of care. The SPACE trial, which focused on patients with inoperable tumors or those declining surgery, was a randomized phase II trial to compare the effectiveness of SBRT and 3DCRT. The SBRT arm received a dose of 66 Gy in three fractions over one week, while the 3DCRT arm underwent treatment with 70 Gy over seven weeks. There were no significant differences in LC and OS between the two arms. Although crude progression rates were slightly higher in the SBRT group (70% vs. 59%), the difference was not statistically significant. Importantly, toxicity in the SBRT arm was lower, with reduced incidences of pneumonitis (19% vs. 34%) and esophagitis (8% vs. 30%) [102]. The only trial that has included a randomization schema between SBRT and HRT was the LUSTRE trial (abstract only)—also mentioned in the section above—the largest phase III study for stage I medically inoperable NSCLC, formally compared SBRT with HRT/CF. There was a trend towards improved local control with SBRT (87.6% vs. 81.2%) but it was not statistically different since the trial lacked sufficient power. Notably, no significant disparities were observed in DFS and OS, and severe late toxicities were rare [94].

RTOG 0236 was a pioneering multi-institutional phase II study that investigated the safety and effectiveness of SBRT in early-stage NSCLC, with updated data released in 2018. The trial involved 55 medically inoperable patients with primary NSCLC and peripherally located tumors, treated with 54 Gy in three fractions. The study reported impressive 3- and 5-year LC rates of 97.6% and 92.7%, respectively, along with a 5-year OS rate of 40%. However, it is worth noting that 27.3% of the patients experienced grade 3 toxicity during the extended follow-up period [103,104]. Another significant study, RTOG 0915, was a randomized phase II comparison of a single fraction of 34 Gy versus four fractions of 12 Gy for peripherally located tumors. In the 5-year report, it was observed that grade ≥3 toxicity occurred in 2.6% of patients who received 34 Gy and 11% of those who received 48 Gy. The 1-year primary control rates were 97% and 93%, while the 5-year rates were 89% and 93% for both groups. Furthermore, the 2-year OS rates were 61% and 78%, and the 5-year were 30% and 41%, with a median survival of 4.1 years and 4.6 years [105,106]. Furthermore, the Roswell Park Cancer Institute conducted a randomized phase study, revealing that a single fraction of 30 Gy was as effective as 60 Gy in three fractions regarding toxicity, LC, and OS [107].

For centrally located tumors, defined variably as those within 2 cm of the proximal bronchial tree and/or abutting the mediastinal pleura, a five-fraction regimen delivered over 2 to 2.5 weeks was better tolerated than a three-fraction regimen using fractions of 10 to 12 Gy, as per the findings of the phase I/II NRG Oncology/RTOG 0813 Trial. The 2-year rates of LC in the 11.5 and 12.0 Gy fraction groups were 89% and 88%, respectively [108].

The revised STARS trial, conducted at the University of Texas MD Anderson Cancer Center, presents long-term results comparing the efficacy of SBRT with surgical treatment for operable early-stage NSCLC. The trial enrolled patients with tumors measuring 3 cm or less in diameter, and SBRT dosing was determined based on tumor location. The study included 80 patients, with a median follow-up of 5.1 years, revealing impressive 3-year and 5-year overall survival rates of 91% (95% CI: 85–98) and 87% (95% CI: 79–95), respectively. Notably, SBRT was well tolerated, with no grade 4–5 toxicities and only a 1% incidence of grade 3 dyspnea, grade 2 pneumonitis, and grade 2 lung fibrosis. Additionally, no serious adverse events were recorded. The propensity-matched video-assisted thoracoscopic surgical lobectomy with mediastinal lymph node dissection (VATS L-MLND) cohort also demonstrated encouraging results, with a 3-year overall survival of 91% (95% CI: 85–98) and a 5-year overall survival of 84% (95% CI: 76–93). Multivariable analysis indicated non-inferiority between SBRT and VATS L-MLND, with a hazard ratio of 0.86 (95% CI: 0.45–1.65, *p* = 0.65). The study provides compelling evidence that long-term survival after SBRT is non-inferior to VATS L-MLND for operable-stage IA NSCLC, making it a promising alternative, especially given its tolerability and equivalent survival outcomes. However, a multidisciplinary approach is strongly recommended to determine the best treatment strategy for individual patients. Definitive conclusions regarding this strategy await randomized trials with appropriate patient recruitment.

Moreover, a comprehensive meta-analysis comparing SBRT with various surgical modalities has provided valuable insights. This analysis reveals that SBRT consistently achieves comparable local control (LC) outcomes, regardless of the surgical approach’s extent. Furthermore, SBRT demonstrates equivalent overall survival (OS) outcomes to surgery in the T1N0M0 subgroup while surpassing sublobar resection. The meta-analysis encompasses data from thirty studies, comprising 29,511 patients, with 17,146 in the surgery group and 12,365 in the SBRT group. The findings clearly favor surgery in terms of 3-year OS and cancer-specific survival (CSS), although no significant difference is observed in 3-year LC. Subgroup analyses further strengthen these findings, with OS in the T1N0M0 subgroup and CSS in the sublobar resection subgroup displaying no notable variances compared to SBRT. While surgery generally yields superior 3-year OS and CSS outcomes, it is important to acknowledge that these results might be influenced by publication bias and heterogeneity. Conversely, SBRT consistently achieves LC outcomes similar to surgery across a range of subgroups. These outcomes underscore the potential clinical advantages of SBRT, which should be thoroughly investigated in future randomized trials [109]. The characteristics of some included trials that assessed hypofractionation and ultra-hypofractionation/SBRT in lung cancer are detailed in Table 6.

#### 3.3.3. Current Guidelines and Consensus Recommendations in NSCLC

According to the guidelines from the European Society for Medical Oncology and the ESTRO-ASTRO Consensus Statement on Practice Recommendations for Lung Cancer Radiotherapy During the COVID-19 Pandemic, HRT is considered suitable for radiotherapy alone or sequential chemoradiation. However, there was a consensus against using hypofractionation in concomitant chemoradiation [110,111].

Regarding SBRT, the ASTRO clinical guidelines, which are also endorsed by the American Society of Clinical Oncology (ASCO), recognize its use for the treatment of stage I NSCLC. For medically inoperable patients with T1-2N0 NSCLC, the recommendations are as follows: caution should be exercised when treating central tumors, and the use of three-fraction regimens should be avoided. To reduce treatment-related toxicity, it is suggested to consider using four or five fractions. Additionally, selecting tumors larger than 5 cm may be suitable for SBRT. Biopsy before SBRT is strongly advised; however, in certain cases, treatment without a tissue diagnosis may be considered with agreement from the multidisciplinary care team. For patients with tumors involving mediastinal structures, SBRT should be approached with caution, and using 4–5 fractions for delivery may help reduce the risks of severe toxicity. For tumors close to the heart or pericardium, SBRT should be delivered in 4–5 fractions, following volumetric and dose constraints from prospective trials with a low incidence of serious toxicities. SBRT may be considered as an option for cT1-2 tumors that abut the chest wall [112,113].

The ESTRO/ACROP consensus recommends risk-adapted fractionation for SBRT. For peripherally located lesions, three fractions of 15 Gy are suggested, while lesions with extensive contact with the chest wall should receive four fractions of 12 Gy each. In cases where patients have no severe comorbidities and are expected to have favorable long-term OS, considering the maximum tolerated dose of three fractions of 18 Gy is advisable [114].

### 3.4. Spine Metastasis

Bone metastases represent a frequent and notable complication in patients with advanced cancer, giving rise to severe and incapacitating effects such as pain, spinal cord compression, hypercalcemia, and pathologic fractures. Notably, around 60% of these metastases affect the spine [115]. The optimal management of these patients requires a comprehensive and collaborative approach, where RT emerges as a highly effective and safe technique for both relieving pain and, in certain cases, achieving long-lasting disease control to improve or prevent neurological compromise [116,117,118,119,120].

When dealing with patients with spinal metastases, initial evaluations of their performance status, overall disease burden, and systemic treatment options play a crucial role. RT can be administered as a standalone treatment, in the postoperative setting, or in conjunction with neuro-interventional procedures. The choice of RT modality, whether CF or HRT, spine stereotactic radiosurgery (SRS), or SBRT, depends on the treatment’s intended purpose and specific circumstances unique to each case [116,120].

#### 3.4.1. Evidence Supporting Moderate Hypofractionation in Spine Metastasis

Spinal metastases are frequently managed with HRT, delivered using diverse fractionation protocols to deliver an EQD2 for tumor control ranging from 30–40 Gy without necessitating prolonged treatment courses. The primary aim of this therapeutic approach is to alleviate pain without excessively burdening a patient with treatment, and results in a partial or complete response in around 50–60% of patients, with a median duration of effect of 4 months [117,119,121].

Multiple prospective randomized trials have demonstrated the equivalence of various dosing schemas, such as 30 Gy in 10 fractions, 24 Gy in 6 fractions, 20 Gy in 5 fractions, and a single 8 Gy fraction, for providing effective pain relief in patients with painful bone metastases [122,123]. According to the Dutch Bone Metastasis study and RTOG 9714, there is no discernible difference in pain control between single- and multiple-fraction regimens for uncomplicated bone metastases [124,125]. However, it is important to note that complicated bone metastases, which include cases with fractures, cord compression, or previous radiotherapy, were excluded from these trials. When there is a risk of pathologic fracture, and these patients are not candidates for surgery, fractionated RT is preferred, as there is a lower fracture risk [124].

A meta-analysis of 29 studies reaffirms the conclusion that both single and multi-fractionated RT show comparable efficacy in patients with spinal metastases in terms of overall response rates, complete response rates, pathological fracture rates, and spinal cord compression rates [119]. Multi-fraction treatment courses demonstrate an 8% re-treatment rate to the same anatomic site due to recurrent pain, in contrast to 20% after a single fraction. However, it is important to highlight that the single-fraction treatment approach optimizes patient and caregiver convenience [117]. The decision regarding the most suitable fractionation should be made through a multidisciplinary approach, considering factors such as performance status, overall disease burden, and systemic treatment options.

#### 3.4.2. Evidence Supporting Ultra-Hypofractionation/SBRT in Spine Metastasis

Spine SRS or SBRT is a highly conformal treatment technique that allows for dose escalation within the treatment volume while minimizing toxicity to adjacent healthy tissues. Typically, spine SRS is delivered in a single treatment, whereas spine SBRT involves 2–5 fractions. The dose distribution is exceedingly conformal, with a rapid dose fall-off to achieve both the ideal dose in the target region and minimal dose to surrounding normal tissues. As a result, margins for the Planning Target Volume (PTV) are restricted to a few millimeters or less [25,126].

SRS and SBRT can be employed in uncomplicated vertebral metastasis, and initial institutional experiences evaluating SRS for vertebral metastases have shown safety and persistent pain relief ranging from 80% to 90%, as well as long-term tumor control when employing different dose regimens [127]. The usual fractionation schemes for the spine include 16 to 18 Gy in one fraction or 24 Gy in one to three fractions, and 27 to 50 Gy in three to five fractions.

The first randomized phase II/III trial (NRG/RTOG 0631) aimed to assess whether patient-reported pain relief was enhanced with SRS compared to HRT for patients with one to three sites of vertebral metastases. Patients in the SRS group received a single dose of 16 or 18 Gy, limited to the involved vertebral level(s) only without including additional spine levels. On the other hand, patients assigned to HRT were treated with 8 Gy to the involved vertebra plus one additional vertebra above and below. Surprisingly, the primary endpoint of pain response at 3 months favored HRT over SRS, with response rates of 60.5% and 41.3%, respectively. The study did not find any significant differences in the occurrence of acute or late adverse effects, vertebral compression fractures, or spinal cord complications at 24 months. Although SRS was not found to be superior for the primary endpoint, there were no spinal cord complications at 2 years post-SRS. This finding may guide further investigation into using spine SRS for oligometastases, where durable cancer control is crucial [128].

In another randomized multicenter phase II/III trial involving 229 patients with painful spine metastases, the comparison between 24 Gy in two fractions of SBRT and 20 Gy in five fractions of HRT demonstrated a significantly superior complete pain response rate of 35% for SBRT compared to 14% for HRT at 3 months (*p* < 0.001), and 32% vs. 16% at 6 months (*p* = 0.004), respectively. This study allowed for the treatment of three consecutive vertebral bodies with SBRT, and both groups demonstrated low toxicity, with a low-risk of grade 2–4 adverse events and no grade 5 adverse events observed [129].

In a large volume of treatment settings, where multilevel or previously irradiated spinal metastases were included, a 30 Gy in a four-fraction scheme was used and reported in 116 patients and 245 treated segments. Remarkably, 54% of the patients had received at least one previous course of radiotherapy, and 31% had undergone previous spine surgery at the treated segment. Despite the population being at an increased risk of toxicity, the 30 Gy in four fractions regimen proved safe and efficacious in 24 months, warranting a randomized clinical trial to further evaluate its potential benefits [130].

An increasing body of evidence from prospective clinical trials supports using SBRT to target oligometastatic lesions, leading to extended progression-free and overall survival in these cases compared to the standard of care [131,132,133]. In the long-term results of the SABR-COMET phase II randomized trial, 99 patients were enrolled with a median follow-up of 51 months. Significantly higher 5-year OS rates were observed with SBRT, reaching 42.3% compared to 17.7% (*p* = 0.006). Importantly, no new grade 2–5 adverse events were reported, and there were no discernible differences in quality of life between the two study arms [133].

These findings further contribute to the growing body of evidence supporting the potential of SABR to improve long-term outcomes in patients with a limited burden of metastatic disease, which could potentially influence treatment decisions. Randomized phase III studies are awaited to understand the benefits and optimal fractionation better.

In LMICs, it is important to acknowledge that resources are often limited, and there may not always be access to the technological and trained personnel necessary to deliver the technique with quality and safety. As a result, we recommend that HRT regimens be preferred when treating spinal metastasis.

## 4. Discussion

Access to RT in LMICs remains a complex and multifaceted challenge. At its core, this issue is marked by a stark contrast between the escalating global burden of cancer and the limited accessibility to RT services within these countries. This discrepancy is concerning, particularly considering the increasing cancer prevalence and the substantial positive impact that radiotherapy can have on patient outcomes.

Hypofractionation is a crucial treatment option in LMICs for several compelling reasons, and its use expanded in the context of the COVID-19 pandemic. Firstly, the economic constraints faced by LMICs necessitate cost-effective solutions. Unlike HICs where labor costs dominate, LMICs struggle with capital and maintenance costs. The availability of equipment remains disproportionately low. Despite being home to 85% of the world’s population, LMICs maintain only 40% of global radiotherapy facilities, leaving a mere 25% of patients with cancer with access to RT [11,134]. Moreover, the shortage of trained healthcare professionals poses a critical barrier to improving access to RT services in LMICs. Technical expertise is vital for high-quality RT, and to cater to the increasing patient needs, human resources must be expanded alongside acquiring additional equipment [3,13].

A comprehensive approach involving close collaboration among international organizations, governments, non-governmental organizations, academic centers, the donor community, and the private sector is imperative. National cancer plans, strong political leadership, and international partnerships are crucial in elevating cancer care standards in LMICs. Establishing partnerships between advanced cancer centers in HICs and clinics in LMICs can offer vital clinical support, education, training, and research initiatives, bridging the gap and bolstering global cancer care endeavors. Investing in radiotherapy, particularly in LMICs, can yield substantial health and economic advantages, signifying a shared global responsibility and a pivotal element for worldwide security and economic progress [3,11,111,135].

Considering these challenges, hypofractionation presents itself as a valuable solution for LMICs. Its reduced treatment time and cost-effectiveness can significantly increase patient access to RT services, and as demonstrated in this review article, without compromising treatment outcomes. Although dependent on a country’s healthcare system, reimbursement often scales with the number of fractions, making fractionation the largest contributing factor to the cost of radiotherapy treatments. A prospective clinical trial assessed the cost-effectiveness of HRT compared to CF for intermediate-risk prostate cancer, finding that HRT was associated with significantly lower costs (€1223 less per patient), potentially reducing national health insurance spending [136]. In another study, the economic impact of employing moderate hypofractionation for localized prostate cancer treatment in the United States was evaluated, with the estimated cost savings ranging from 25% to 50% [137]. In LMICs, a study estimated potential savings of $1.1 billion USD for breast cancer and $606 million USD for prostate cancer treatments between 2019 and 2025 [10]. These studies highlight the cost-saving potential of HRT across diverse settings and emphasize the economic impact of different fractionation schemes [16].

There is an ongoing reluctance to adopt hypofractionation in some LMICs, influenced by various factors, including economic concerns related to reimbursement models, fears of treatment-related toxicities, the need for more long-term clinical evidence, geographical variability in practice, lack of awareness and education, patient-specific factors such as breast reconstruction type, and regulatory or institutional barriers [138]. Potential solutions include revising reimbursement structures, improving education and awareness for healthcare professionals, focusing on long-term treatment outcomes and safety data, promoting interdisciplinary collaboration, and creating guidelines or recommendations to encourage the broader adoption of hypofractionation. Based on the best available evidence, this study can provide valuable insights for physicians when making decisions about applying hypofractionation regimens in clinical practice.

## 5. Conclusions

The importance of HRT in LMICs cannot be overstated. By embracing hypofractionation and investing in advanced technologies to support its safe and effective implementation, LMICs can bridge the gap in RT availability and improve the survival rates and quality of life for their cancer patients. It is crucial for the global community to collaborate and support LMICs in their efforts to provide equitable and effective cancer care for all.

## Figures and Tables

**Table 1:** Moderate hypofractionation—ductal carcinoma in situ (DCIS).

Trial	Sample Size	Inclusion Criteria	RT Technique	Fractionation	Boost	Outcomes	Median Follow-Up	Results
BIG 3-07 TROG 07.01 [54]	1608	Non-low-risk DCIS with 1 mm margins	2D or 3D	C-WBI: 50 Gy in 25 fx. H-WBI: 42.5 Gy in 16 fx	16 Gy in 8 fx was given if allocated	LR	6.6 years	5 yr FFLR: 97.1% (Boost) vs. 92.7% (No Boost), HR: 0.47 (95% CI: 0.31–0.72, *p* < 0.001) Grade ≥ 2 Breast Pain: 14% (Boost) vs. 10% (No Boost) Grade ≥ 2 Induration: 14% (Boost) vs. 6% (No Boost) 5 yr IBTR: 6% (Both Arms, Non-inferior)

Legend: DCIS: ductal carcinoma in situ; C-WBI: conventional whole-breast irradiation; fx: fractions; H-WBI: hypofractionated whole-breast irradiation; LR: local recurrence; FFLR: free from local recurrence.

**Table 2:** Moderate hypofractionation—invasive breast cancer.

Trial	Sample Size	Inclusion Criteria	RT Technique	Fractionation	Boost	Outcomes	Median Follow-Up	Results
OCOG [42,44]	1234	pT1-2 post lumpectomy	2D (including cobalt) and 3D	42.5 Gy in 16 fx 50 Gy in 25 fx	No	LRIC; DR, BC, LRT.	5.9 years	5 yr LRIC: 97.2% (Short Arm), 96.8% (Long Arm), Abs Difference: 0.4% (95% CI: −1.5% to 2.4%) 3 yr BC: 76.8% (Short Arm), 77.0% (Long Arm), Abs Difference: −0.6% (95% CI: −6.5% to 5.5%)
START A [41] and B [43]	START-A 2236 START-B 2215	pT1-3a pN0-1 M0) requiring radiotherapy after primary BCS or mastectomy, with clear tumor margins ≥1 mm	2D (including cobalt) and 3D	START-A: f 50 Gy in 25 fx over 5 weeks with 41.6 Gy or 39 Gy in 13 fx START-B: 50 Gy in 25 fx with 40 Gy in 15 fx	Yes in 42.6% with 10Gy	LR and LRT	9.3 years for START-A, and 9.9 years for START-B.	START-A: 10 yr LR: 6.3% (41.6 Gy), 7.4% (50 Gy, HR 0.91, *p* = 0.65), 8.8% (39 Gy, HR 1.18, *p* = 0.41) START-B: 10 yr LR: 4.3% (40 Gy), 5.5% (50 Gy, HR 0.77, *p* = 0.21)
DBCG HYPO [52]	1854	>40 years of age after BCS for node-negative breast cancer or DCIS (13%)	3D	50 Gy in 25 fx 40 Gy in 15 fx	Yes, 23.1% received 10 Gy as boost	BI and LR	7.26 years	3 yr BI: 11.8% (50 Gy), 9.0% (40 Gy), risk difference: −2.7% (*p* = 0.07) 9 yr LRR: 3.3% (50 Gy), 3.0% (40 Gy) 9 yr OS: 93.4% (both 50 Gy and 40 Gy)
Peking Union Medical College, Beijing, China [46]	820	Post-mastectomy pT3-4 N+ (at least 4 nodes)	2D	50 Gy in 25 fx 43.5 Gy in 15 fx Including chest wall and nodal irradiation	No	5-year LRR	5 years	5-year LRR: 8.3% (hypofractionated) vs. 8.1% (conventional), HR 1.10, 90% CI 0.72 to 1.69, *p* < 0.0001

Legend: LRIC: local recurrence of invasive cancer; LRR: locoregional recurrence; DFS: disease-free survival; DR: distant recurrence; BC: breast cosmesis; LRT: late radiation toxicity; BI: breast induration; LR: local-regional tumour relapse; OS: overall survival; BCS: breast cancer-specific survival; BCS: BCS surgery; fx: fractions.

**Table 3:** Ultra-hypofractionation—invasive breast cancer.

Trial	Sample Size	Inclusion Criteria	RT Technique	Fractionation	Boost	Outcomes	Median Follow-Up	Results
FAST Trial [56]	915	pT1–2 pN0 after BCS	3D/IMRT	50 Gy in 25 fx of 2.0 Gy 30 Gy in 5 once-weekly fx of 6.0 Gy 28.5 Gy in 5 once-weekly fx of 5.7 Gy.	No	Breast appearance at 2 and 5 years; physician assessments NTE and LR	9.9 years	NTE 1.64 (95% CI, 1.08 to 2.49; *p* = 0.019) for 30 Gy and 1.10 (95% CI, 0.70 to 1.71; *p* = 0.686) for 28.5 Gy versus 50 Gy
FAST FORWARD [57]	4096	Invasive carcinoma of the breast (pT1–3, pN0–1)	3D/IMRT	40 Gy/15 fx 27 Gy/5 fx daily 26 Gy/5 fx daily	Yes, 24.3% received 10–16 Gy in 2Gy/fx	5y-IBTR, NTE, LRR and DM	6 years	40 Gy vs. 26 Gy 5 yr IBTR 2.3% vs. 2.0% vs. 1.5%, non-inferior 5 yr LRR 3.2% vs. 2.6% vs. 2.1%, non-inferior 5 yr DM 4.3% vs. 5.0% vs. 5.6%, non-inferior Patient and photographic assessments showed higher NTE risk for 27 Gy

Legend: LRR: locoregional relapse; DM: distant metastasis; NTE: normal tissue effect; IBTR: in breast tumor recurrence; fx: fractions.

**Table 4:** Hypofractionation for prostate.

Trial	Sample Size	Inclusion Criteria	RT Technique	Fractionation	Outcomes	Median Follow-Up	Results
PROFIT [79]	1206	Intermediate-risk	3D/IMRT	78 Gy/39 fx 60 Gy/20 fx	BCF	6 years	5-year BCF DFS was 85% in both arms (HR [short v standard], 0.96; 90% CI, 0.77 to 1.2)
CHHIP [76,78]	3216	Localized prostate cancer (pT1b–T3N0) Mostly intermediate 73%	3D/IMRT	74 Gy/37 fx 60 Gy/20 fx 57 Gy/19 fx ADT allowed	Time to biochemical or clinical failure	5.2 years	5 yr BCF 74 Gy: 88.3% failure-free at 5 years. 60 Gy: 90.6%, HR vs. 74 Gy: 0.84, pNI = 0.0018. 57 Gy: 85.9%, HR vs. 74 Gy: 1.20, pNI = 0.48.

Legend: BCF: biochemical–clinical failure; HR: hazard ratio; fx: fractions; DFS: disease-free survival.

**Table 5:** Ultra-hypofractionation and SBRT for prostate.

Trial	Sample Size	Inclusion Criteria	RT Technique	Fractionation	Outcomes	Median Follow-Up	Results
HYPO-RT-PC [83,84]	1200	Intermediate-to-high-risk prostate cancer: T1c–T3a with 1–2 factors: stage T3a, Gleason ≥7, PSA 10–20 ng/mL; no lymph node/metastases involvement	3D/IMRT/VMAT Image-guided	42.7 Gy/7 fx 78 Gy/39 fx	FFS and QOL	4 years	5 yr FFS 84% in both arms Urinary bother: conventional 33% (43/132) vs. ultra-hypofractionation 28% (33/120), *p* = 0.38. Bowel bother: conventional 33% (43/129) vs. ultra-hypofractionation 28% (34/123), *p* = 0.33. Sexual bother: conventional 60% (75/126) vs. ultra-hypofractionation 50% (59/117), *p* = 0.15. Global health/QOL: conventional 42% (56/134) vs. ultra-hypofractionation 37% (46/125), *p* = 0.41.
PACE-B [85,86]	874	Low- and intermediate-risk (91% were intermediate-risk, 9% low)		78 Gy/39 fx or 62 Gy/20 fx 36.25 Gy to PTV, 40 Gy to CTV	BCF	6.1 years	5-year BCF event-free rate: CRT 94.6% (91.9–96.4%) vs. SBRT 95.7% (93.2–97.3%). SBRT non-inferior to CRT: HR = 0.74 (0.47–1.17), *p*-value = 0.007. Absolute difference at 5 years: 1.36% (90% CI: 0.87–2.80%). Toxicity at 5 years: RTOG G2+ GU: CRT 3.2% (11/348) vs. SBRT 5.5% (20/363), *p* = 0.14. RTOG G2+ GI: both groups had 1 case (CRT 1/348, SBRT 1/363), *p* = 0.99.

Legend: QOL: quality of life; HR: hazard ratio; fx: fractions; CRT: conventional radiotherapy. GI: gastrointestinal; GU: genitourinary.

**Table 6:** Hypofractionation for lung NSCLC.

Trial	Sample Size	Inclusion Criteria	RT Technique	Fractionation	Outcomes	Median Follow-Up	Results
SOCCAR [98]	130	Stage III	3D/IMRT 4D CT allowed	55Gy/20 fx Concurrent vs. sequential chemo	TRTM	2.93 years	TRTM 2.9% (CI 0.36–10.2%) concurrent vs. 1.7% (CI 0.043–9.1%) sequential; RR 1.25 (CI 0.55, 2.84)
CHISEL [93]	101	Stage I/II, inoperable or refusing surgery	3D	SBRT 54 Gy/3 fx 48 Gy/4 fx 66 Gy/33 fx, or 50 Gy/20 fx	Time to local treatment failure	2.1 years	Local progression: 14% SABR vs. 31% standard radiotherapy; SABR had improved FFF (HR 0.32, *p* = 0.0077)
RTOG 0915 [105,106]	94	Stage I/II peripheral inoperable	IMRT 4D CT allowed	34 Gy/1 fraction 48 Gy/4 fx	Rate of grade 3 or higher	4 years	Toxicity rates (Grade 3+): 34 Gy (Arm 1): 2.6% 48 Gy (Arm 2): 11.1% Primary tumor failure: 34 Gy: 10.6% (CI: 3.3–23.1%) vs. 48 Gy: 6.8% (CI: 1.7–16.9%) OS: 34 Gy: 29.6% (CI: 16.2–44.4%) vs. 48 Gy: 41.1% (CI: 26.6–55.1%)

Legend: TRTM: treatment-related mortality; FFF: freedom from failure.
